# Real-world efficacy and safety of TACE plus camrelizumab and apatinib in patients with HCC (CHANCE2211): a propensity score matching study

**DOI:** 10.1007/s00330-023-09754-2

**Published:** 2023-06-27

**Authors:** Zhi-Cheng Jin, Bin-Yan Zhong, Jian-Jian Chen, Hai-Dong Zhu, Jun-Hui Sun, Guo-Wen Yin, Nai-Jian Ge, Biao Luo, Wen-Bin Ding, Wen-Hui Li, Li Chen, Yu-Qing Wang, Xiao-Li Zhu, Wei-Zhu Yang, Hai-Liang Li, Gao-Jun Teng, for the CHANCE Investigators

**Affiliations:** 1 Center of Interventional Radiology & Vascular Surgery, Department of Radiology, Zhongda Hospital, Medical School, Southeast University, Nanjing, 210009 China; 2 Department of Interventional Radiology, The First Affiliated Hospital of Soochow University, Soochow University, Suzhou, 215006 China; 3 Hepatobiliary and Pancreatic Interventional Treatment Center, Division of Hepatobiliary and Pancreatic Surgery, The First Affiliated Hospital, Zhejiang University School of Medicine, Hangzhou, 310003 China; 4 Department of Interventional Radiology, Jiangsu Cancer Hospital & Jiangsu Institute of Cancer Research & The Affiliated Cancer Hospital of Nanjing Medical University, Nanjing, 210009 China; 5 Department of Interventional Radiology, Eastern Hospital of Hepatobiliary Surgery, Second Military Medical University, Shanghai, 200438 China; 6 Department of Interventional Radiology, Nantong First People’s Hospital, Nantong, 226001 China; 7 Department of Interventional Radiology, Yancheng Third People’s Hospital, Yancheng, 224008 China; 8 Department of Interventional Radiology, Union Hospital of Fujian Medical University, Fuzhou, 350001 China; 9 Department of Minimally Invasive Intervention, The Affiliated Cancer Hospital of Zhengzhou University, Zhengzhou, 450008 China

**Keywords:** Carcinoma, hepatocellular, Chemoembolization, therapeutic, Immunotherapy, Molecular targeted therapy, Combined modality therapy

## Abstract

**Objectives:**

This study aimed to investigate the efficacy and safety of transarterial chemoembolization (TACE) plus camrelizumab, a monoclonal antibody targeting programmed death-1, and apatinib for patients with intermediate and advanced hepatocellular carcinoma (HCC) in a real-world setting.

**Methods:**

A total of 586 HCC patients treated with either TACE plus camrelizumab and apatinib (combination group, *n* = 107) or TACE monotherapy (monotherapy group, *n* = 479) were included retrospectively. Propensity score matching analysis was used to match patients. The overall survival (OS), progression-free survival (PFS), objective response rate (ORR), and safety in the combination group were described in comparison to monotherapy.

**Results:**

After propensity score matching (1:2), 84 patients in the combination group were matched to 147 patients in the monotherapy group. The median age was 57 years and 71/84 (84.5%) patients were male in the combination group, while the median age was 57 years with 127/147 (86.4%) male in the monotherapy group. The median OS, PFS, and ORR in the combination group were significantly higher than those in the monotherapy group (median OS, 24.1 vs. 15.7 months, *p* = 0.008; median PFS, 13.5 vs. 7.7 months, *p* = 0.003; ORR, 59.5% [50/84] vs. 37.4% [55/147], *p* = 0.002). On multivariable Cox regression, combination therapy was associated with significantly better OS (adjusted hazard ratio [HR], 0.41; 95% confidence interval [CI], 0.26–0.64; *p* < 0.001) and PFS (adjusted HR, 0.52; 95% CI, 0.37–0.74; *p* < 0.001). Grade 3 or 4 adverse events occurred in 14/84 (16.7%) and 12/147 (8.2%) in the combination and monotherapy groups, respectively.

**Conclusions:**

TACE plus camrelizumab and apatinib showed significantly better OS, PFS, and ORR versus TACE monotherapy for predominantly advanced HCC.

**Clinical relevance statement:**

Compared with TACE monotherapy, TACE plus immunotherapy and molecular targeted therapy showed better clinical efficacy for predominantly advanced HCC patients, with a higher incidence of adverse events.

**Key Points:**

*• This propensity score–matched study demonstrates that TACE plus immunotherapy and molecular targeted therapy have a longer OS, PFS, and ORR compared with TACE monotherapy in HCC.*

*• Grade 3 or 4 adverse events occurred in 14/84 (16.7%) patients treated with TACE plus immunotherapy and molecular targeted therapy compared with 12/147 (8.2%) patients in the monotherapy group, while no grade 5 adverse events were observed in all cohorts.*

**Supplementary information:**

The online version contains supplementary material available at 10.1007/s00330-023-09754-2.

## Introduction

Hepatocellular carcinoma (HCC) is the sixth most commonly diagnosed cancer and the third leading cause of cancer-related mortality worldwide [1]. Although curative surgical therapies can yield long-term survival at an early stage, many patients are not appropriate candidates [2, 3]. Transarterial chemoembolization (TACE) is recommended as standard therapy for intermediate-stage HCC according to the current guidelines and is also the most widely used in advanced HCC in real-world practice [3, 4]. Several RCTs that combined TACE with the systemic drug failed to provide beneficial overall survival (OS), although median progression-free survival (PFS) was significantly prolonged (~ 12 months) with TACE plus sorafenib than TACE alone reported by TACTICS trial [3, 5–7].

Recently, immunotherapy with checkpoint inhibitors has shown strong anti-tumor activity and the combination of programmed death (PD)-(ligand [L]) 1 inhibitor and antiangiogenic molecular target drugs have become promising therapeutics for HCC since the favorable results of the IMbrave150 and ORIENT-32 trials [8–10]. A humanized PD-1 monoclonal antibody within immunotherapy, camrelizumab (SHR-1210) showed good tolerance and antitumor effect on a variety of solid tumors, including HCC [11, 12]. Apatinib is a small-molecule tyrosine kinase inhibitor with highly selective for vascular endothelial growth factor receptor-2 and exhibits potential survival benefits for Chinese patients with advanced HCC [13]. Based on these results, both camrelizumab and apatinib were recently approved by the National Medical Products Administration of China (NMPA) for advanced HCC [14]. The clinical efficacy and safety in advanced HCC patients of camrelizumab plus apatinib were reported in phase II RESCUE trial [15]. Camrelizumab plus apatinib lead to a 45.7% objective response with a median PFS of 5.7 months in the first-line setting, and 25.0% with 5.5 months in the second-line setting. Recently, the phase III trial using camrelizumab plus apatinib was announced to provide a statistically significant survival benefit versus sorafenib in the first line and presented at the European Society of Medical Oncology (ESMO) Congress 2022 [16].

There is a rationale supporting combining TACE plus anti-PD-(L)1 and molecular targeted therapies. The immunosuppressive tumor microenvironment of HCC promoted by the tumor cells and the infiltrating stromal and immune cells generally correlates with a worse prognosis [8, 17, 18]. TACE induces immunogenic tumor cell death and tumor-specific immune responses with the release of tumoral antigens, proinflammatory cytokines, vascular endothelial growth factor, and hypoxia-inducible factor-1α [18–21]. It could act by turning the immunosuppressive “cold tumor” into a “hot tumor” for HCC and provides a potential synergistic anti-tumor effect by combing TACE with anti-PD-(L)1 and molecular targeted therapies [18, 19, 22]. Thus, whether TACE plus camrelizumab and apatinib could effectively leverage the synergistic benefits without prominent increasing toxicity remain unknown.

In this multicenter, retrospective cohort study, we aimed to evaluate the efficacy and safety of TACE plus camrelizumab and apatinib for patients with intermediate and advanced HCC in a real-world setting.

## Materials and methods

### Study design and participants

The multicenter, retrospective, cohort study included patients with intermediate or advanced HCC treated with TACE plus camrelizumab and apatinib (combination group) or TACE alone (monotherapy group) between January 2018 and May 2021 from a nationwide registry in China. This study was approved by the institutional review board of Clinical Research of Zhongda Hospital, affiliated with Southeast University (Ethics Approval ID: 2021ZDSYLL179-P01), and written informed consent was waived. The study was conducted per the Declaration of Helsinki and registered at www.clinicaltrials.gov (NCT04975932). The Strengthening the Reporting of Observational Studies in Epidemiology (STROBE) statement for the observational cohort was followed. The study included patients from the subset of the CHANCE001 study [23] and subsequent expansion cohorts, and all data were derived from the database of the national registry platform entitled “Chinese Liver Cancer Clinical Study Alliance (CHANCE)” sponsored by the Chinese College of Interventionalists (CCI). All patients included in this study were not enrolled in those industry-sponsored clinical trials.

The following inclusion criteria were set: (1) patients with a diagnosis that was histologically, cytologically, or clinically confirmed per the American Association for the Study of Liver Disease criteria or the European Association for the Study of the Liver criteria [24, 25]; (2) patients  ≥ 18 years old; (3) Barcelona Clinic Liver Cancer (BCLC) stage B or C; (4) Child-Pugh grade A or B without uncontrollable ascites or hepatic encephalopathy; (5) Eastern Cooperative Oncology Group (ECOG) performance status 0–1; (6) patients received the combination therapy (i.e., TACE plus camrelizumab and apatinib) or TACE monotherapy during the same period. The timeframe criteria of combination therapy were defined as administration of TACE concurrently with or up to 60 days before camrelizumab, and apatinib was concomitant with TACE or camrelizumab. At least one cycle of camrelizumab agent should be used after the TACE procedure. Patients with incomplete clinical or follow-up data were excluded.

The treatment decision-making for patients is either using TACE alone or TACE combined with systemic agents based on individual circumstances and physician discretion. Multidisciplinary teams in the participating hospitals for HCC dominated the treatment decision following the BCLC guidelines or China National Liver Cancer guidelines. Physicians would let the patient and his/her family members know the advantages and disadvantages of the different treatment protocols, including potential treatment outcomes, financial burden, and treatment-related complications before the final decision-making.

### Procedures

Patients included in the study received conventional TACE (cTACE) or drug-eluting beads TACE (DEB-TACE) that were performed by interventional radiologists with at least 10 years of experience from participating centers. All the TACE procedures were applied according to TACE standardization (details are given in the supplementary appendix on page 2) [26, 27]. Chemotherapeutic drugs including doxorubicin, epirubicin, and others could be selected for both cTACE and DEB-TACE according to the clinical practice of the participating centers. Subsequent TACE procedures were done “on demand” when unsatisfactory tumor necrosis, local recurrence, or new intrahepatic lesions was suspected based on follow-up contrast-enhanced computed tomography or magnetic resonance imaging. TACE was discontinued in cases of deterioration of liver function to Child-Pugh C (uncontrollable ascites, severe jaundice, overt hepatic encephalopathy, or hepatorenal syndrome), ECOG performance status  > 2, or continuous progression of target lesions after three TACE sessions according to the clinical practice of the participating centers.

Patients received intravenous camrelizumab at doses of 200 mg for 20–60 min every 3 weeks and oral apatinib 250 mg once per day in a combination group. Temporary camrelizumab interruption was allowed because of toxicities but dose reduction was not allowed. Dose reduction for apatinib because of toxicities was allowed. Drugs were discontinued in the event of disease progression, unacceptable toxic effects, patient choice, or the recommendation of the physicians.

### Follow-up and assessments

Standardized patient assessments were arranged before every treatment session (both for TACE and camrelizumab) or during every routine follow-up at a minimum of 3–4 weeks intervals. Tumor response on all follow-up cross-sectional computed tomography or magnetic resonance imaging scans was determined based on modified Response Evaluation Criteria in Solid Tumors (mRECIST) by two independent radiologists with more than five years of experience at each participating center. The senior radiologists made the final decision in case of any disagreement. All radiologists who participated in this study received lecture-based and online instruction training to standardize tumor response evaluation.

Safety assessments were done continuously through routine laboratory tests and vital signs. Adverse events were analyzed as treatment-emergent adverse events and mostly graded at the time of events; this approach was consistent across all participating centers. The severity of adverse events was assessed according to the National Cancer Institute Common Terminology Criteria for Adverse Events (CTCAE; version 5.0). All patients received routine follow-ups until death or the end of the study (May 30, 2022).

### Outcomes

The outcome measure was OS, defined as the interval from the date of enrollment time to the date of all-cause death. Additional outcome measures included PFS, objective response rate (ORR), and safety. PFS was defined as the interval from the date of enrollment time of two groups to the date of disease progression or all-cause death. ORR was defined as the proportion of patients with a partial or complete response to treatment according to mRECIST. For patients treated with systemic therapy after TACE in the combination group or patients in the TACE monotherapy group, the enrollment time was defined as the date of the initial TACE procedure during the study period. For patients treated with systemic therapy before TACE, the enrollment time was defined as the date of initiation of systemic therapy.

### Statistical analysis

To reduce the potential confounding and selection bias, propensity score matching (PSM) analysis was performed and 1:2 nearest-neighbor matching without replacement using a caliper width of 0.05 was set. Propensity scores were calculated using logistic regression models with the following preplanned covariates: sex, age, ECOG performance status, hepatitis B virus (HBV) infection (absent vs. present), cirrhosis (absent vs. present), Child-Pugh grade (A vs. B), six-to-twelve criteria (≤ 6,  > 6 and  ≤ 12,  > 12), BCLC stage (B vs. C), extrahepatic spread (absent vs. present), macroscopic vein invasion (absent vs. present), and HCC-related treatment history (absent vs. present). The standardized mean difference was used to evaluate the covariate balance for the propensity-matched cohorts (Fig. S1). Four sensitivity analyses were performed to assess the robustness of the PSM analysis (detailed in the appendix pages 3–4).

To compare patient baseline characteristics between the two groups, the Mann-Whitney U test (nonnormally distributed data) or Student’s t-test (normally distributed data) was employed to analyze continuous variables, and the chi-squared test or Fisher exact test was employed to compare categorical variables. Kaplan-Meier method estimates and the log-rank test were used to compare PFS and OS between the two groups. Univariable and multivariable Cox proportional hazards models were used to evaluate the independent effect of combination therapy on PFS and OS based on the propensity-matched sample and unmatched all patients. The multivariable analysis with forwarding procedure includes the variables with *p* value  <  = 0.1 from the univariable analysis. Subgroup analysis comparing PFS and OS between two groups was performed for prespecified clinically relevant parameters, including ECOG performance status, HBV infection, cirrhosis, Child-Pugh grade, six-to-twelve criteria [28], BCLC stage, extrahepatic spread, macroscopic vein invasion, HCC-related treatment history, and previous TACE history. Six-to-twelve criteria was a prognostic model presented as the sum of tumor size and number that can stratify the prognosis of TACE patients with cut-off values of 6 and 12 [28].

Considering the sample size calculation was limited due to the retrospective nature of this study, the power calculation was carried out for the present study (supplementary appendix page 4). A 2-tailed *p* value of  < 0.05 was considered statistically significant. All the above statistical analyses were performed using R (version 4.1.0; R Project for Statistical Computing, http://www.r-project.org) with MatchIt [29], survival [30], and ipw [31] packages, and SPSS (version 24.0; IBM).

## Result

### Patient characteristics

**Fig. 1:**
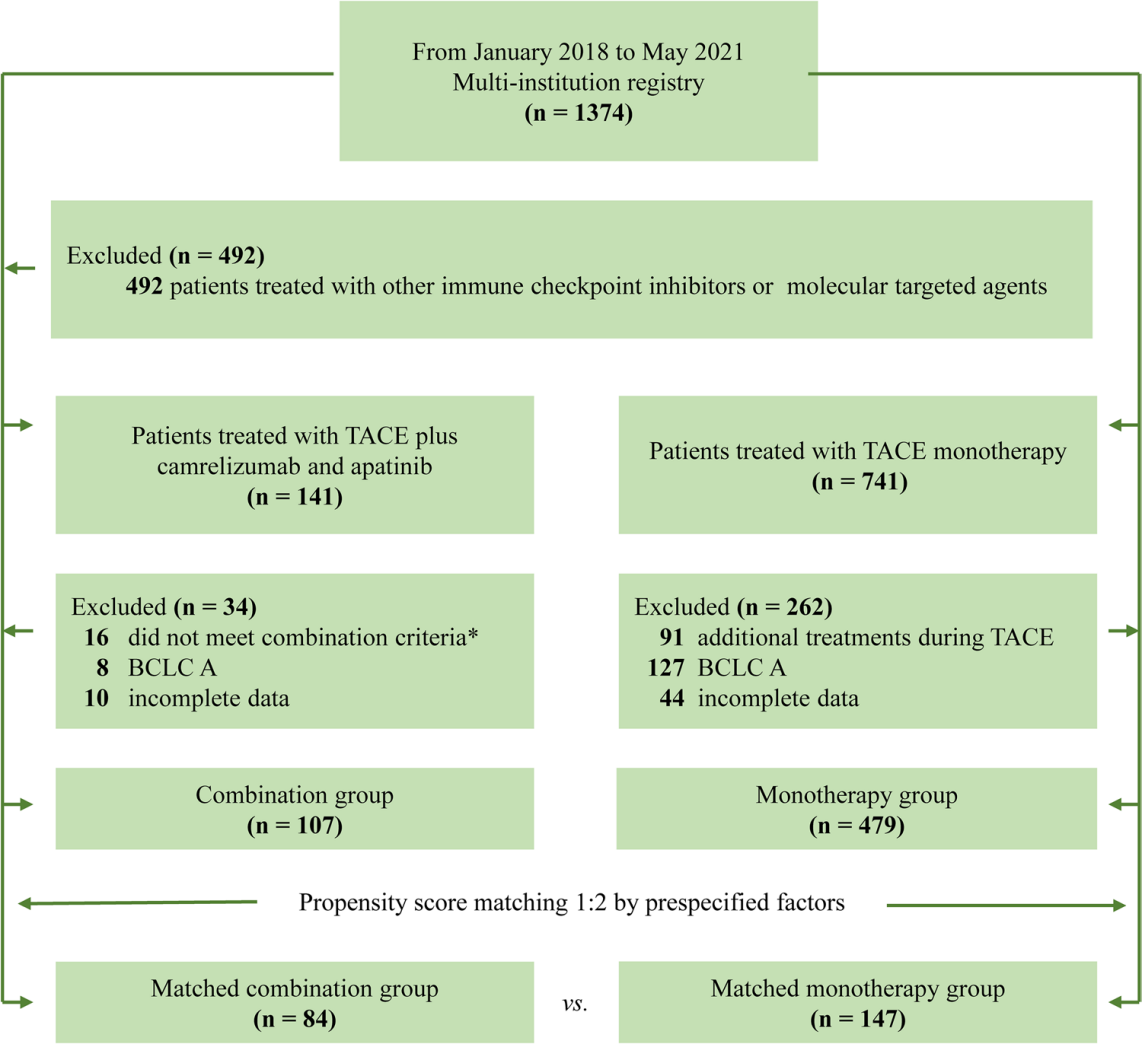
Patient selection flowchart. TACE, transarterial chemoembolization; BCLC, Barcelona Clinic Liver Cancer. ^#^The timeframe criteria of combination therapy were defined as administration of TACE concurrently with or up to 60 days before camrelizumab, and apatinib was concomitant with TACE or camrelizumab

During the study period, a total of 586 patients were included from the nationwide registry, of whom 107 patients received TACE plus camrelizumab and apatinib in the combination group and 479 patients treated with TACE monotherapy. Among them, patients treated with TACE plus camrelizumab and apatinib came from 17 centers, while patients treated with TACE monotherapy came from 43 centers. Figure 1 shows the patient selection flowchart of the study. The median age was 54 years (interquartile range [IQR], 48–64) and 91 (85%) of 107 patients were male in the combination group, while the median age was 61 years (54–68) with 401/479 (83.7%) male in the monotherapy group. Before the matching, the combination group had more patients with advanced HCC, while around half of the patients received prior HCC-related treatment. After propensity score matching (1:2), 84 patients in the combination group were matched to 147 patients in the monotherapy group. No significant differences in baseline characteristics were observed in matched cohorts (Table 1). The median age was 57 years (IQR, 49–64), and 71 (84.5%) of 84 patients were male in the combination group, while the median age was 57 years (51–66) with 127/147 (86.4%) male in the monotherapy group. The number of advanced HCC patients was 56/84 (66.7%) and 95/147 (64.6%) in each group, respectively. In the propensity score matched data set, the median follow-up time for patients was 20.0 months in the combination group and 16.6 months in the monotherapy group (*p* = 0.200). During the follow-up, the median numbers of camrelizumab and TACE in combination group were four cycles (IQR, 3–7 cycles) and three times (IQR, 2–4 times), respectively, and the median number of TACE in monotherapy was three times (IQR, 2–6 times).

**Table 1:** Patient baseline characteristics of monotherapy and combination groups before and after PSM

Characteristics^*^	Before PSM			After PSM		
	Monotherapy group (*n* = 479)	Combination group (*n* = 107)	*p* value	Monotherapy group (*n* = 147)	Combination group (*n* = 84)	*p* value
Median age (years)^#^	61 (54–68)	54 (48–64)	< 0.001	57 (51–66)	57 (49–64)	0.416
Sex			0.847			0.845
Female	78 (16.3)	16 (15.0)		20 (13.6)	13 (15.5)	
Male	401 (83.7)	91 (85.0)		127 (86.4)	71 (84.5)	
Etiology			0.109			0.616
Hepatitis B virus	356 (74.3)	88 (82.2)		108 (73.5)	65 (77.4)	
Others	123 (25.7)	19 (17.8)		39 (26.5)	19 (22.6)	
Cirrhosis			0.028			> 0.999
Absent	182 (38.0)	28 (26.2)		39 (26.5)	22 (26.2)	
Present	297 (62.0)	79 (73.8)		108 (73.5)	62 (73.8)	
Child-Pugh class			> 0.999			0.732
A	409 (85.4)	91 (85.0)		128 (87.1)	71 (84.5)	
B	70 (14.6)	16 (15.0)		19 (12.9)	13 (15.5)	
ECOG PS			0.007			0.732
0	386 (80.6)	73 (68.2)		106 (72.1)	58 (69.0)	
1	93 (19.4)	34 (31.8)		41 (27.9)	26 (31.0)	
BCLC stage			< 0.001			0.865
B	233 (48.6)	31 (29.0)		52 (35.4)	28 (33.3)	
C	246 (51.4)	76 (71.0)		95 (64.6)	56 (66.7)	
Tumor number			0.147			0.352
1	101 (21.1)	17 (15.9)		34 (23.1)	15 (17.9)	
2	99 (20.7)	16 (15.0)		27 (18.4)	10 (11.9)	
3	47 (9.8)	16 (15.0)		16 (10.9)	11 (13.1)	
> 3	232 (48.4)	58 (54.2)		70 (47.6)	48 (57.1)	
Largest tumor diameter			0.681			0.433
≤ 5	174 (36.3)	36 (33.6)		58 (39.5)	28 (33.3)	
> 5	305 (63.7)	71 (66.4)		89 (60.5)	56 (66.7)	
Six-and-twelve			0.803			0.904
≤ 6	70 (14.6)	13 (12.1)		24 (16.3)	12 (14.3)	
> 6 and ≤ 12	234 (48.9)	54 (50.5)		65 (44.2)	39 (46.4)	
> 12	175 (36.5)	40 (37.4)		58 (39.5)	33 (39.3)	
Macroscopic vein invasion			0.143			> 0.999
Absent	286 (59.7)	55 (51.4)		78 (53.1)	45 (53.6)	
Present	193 (40.3)	52 (48.6)		69 (46.9)	39 (46.4)	
Extrahepatic spread			< 0.001			0.888
Absent	369 (77.0)	60 (56.1)		90 (61.2)	53 (63.1)	
Present	110 (23.0)	47 (43.9)		57 (38.8)	31 (36.9)	
TACE type			0.040			0.821
cTACE	345 (72.0)	88 (82.2)		114 (77.6)	67 (79.8)	
DEB-TACE	134 (28.0)	19 (17.8)		33 (22.4)	17 (20.2)	
HCC-related treatment history			< 0.001			0.639
Absent	398 (83.1)	48 (44.9)		90 (61.2)	48 (57.1)	
Present	81 (16.9)	59 (55.1)		57 (38.8)	36 (42.9)	
Surgery	35 (7.3)	22 (20.6)	< 0.001	22 (15.0)	13 (15.5)	> 0.999
TACE	54 (11.3)	47 (43.9)	< 0.001	40 (27.2)	29 (34.5)	0.821
Ablation	19 (4.0)	16 (15.0)	< 0.001	16 (10.9)	12 (14.3)	0.581
Radiotherapy	10 (2.1)	11 (10.3)	< 0.001	9 (6.1)	7 (8.3)	0.713

^*^ Except where indicated, data are number (%). Chi-squared test or Fisher exact test for categorical variables were applied

^#^ Data were continuous variables, expressed in median (interquartile range), and were compared by using the Mann-Whitney U test

*PSM*, propensity score matching; *ECOG PS*, Eastern Cooperative Oncology Group performance status; *BCLC*, Barcelona Clinic Liver Cancer; *TACE*, conventional transarterial chemoembolization; *cTACE*, conventional TACE; *DEB-TACE*, drug-eluting beads TACE; *HCC*, hepatocellular carcinoma

### Efficacy

**Fig. 2:**
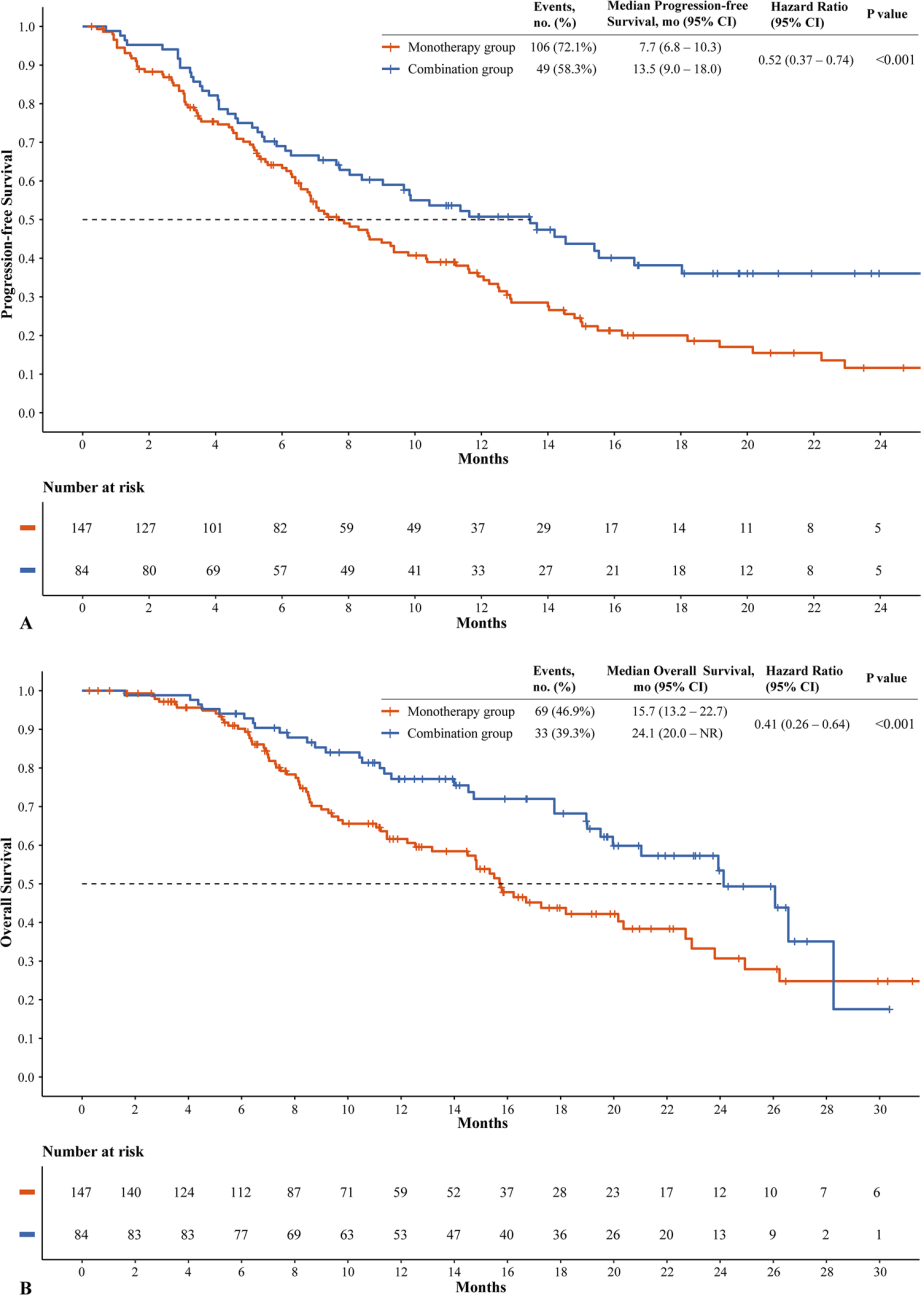
Kaplan–Meier curves of progression-free survival (**A**) and overall survival (**B**) after matching

A total of 49 (58.3%) of 84 patients in the combination group and 106 (72.1%) of 147 patients in the monotherapy group had disease progression or died. Median PFS and OS were significantly longer with combination therapy than with monotherapy (for median PFS, 13.5 months [95% CI, 9.0–18.0] vs. 7.7 months [95% CI, 6.8–10.3], *p* = 0.003; for median OS, 24.1 months [95% CI, 20.0–NR] vs. 15.7 [95% CI, 13.2–22.7] months, *p* = 0.008) (Fig. 2). Patients in the combination group achieved a higher ORR than those in the monotherapy group (59.5% [50/84] vs. 37.4% [55/147], respectively; *p* = 0.002). Before matching, the median PFS, OS, and ORR in the combination group were significantly higher than those in the monotherapy group (PFS, 11.6 [95% CI, 9.7–15.5] months vs. 8.9 [95% CI, 7.8–9.9] months, *p* = 0.005; OS, 24.1 [95% CI, 19.5–NR] months vs. 16.9 [95% CI, 15.5–19.1] months, *p* = 0.003; ORR, 55.1% [59/107] vs. 39.2% [188/479], *p* = 0.004; Fig. S2 and 3).

**Fig. 3:**
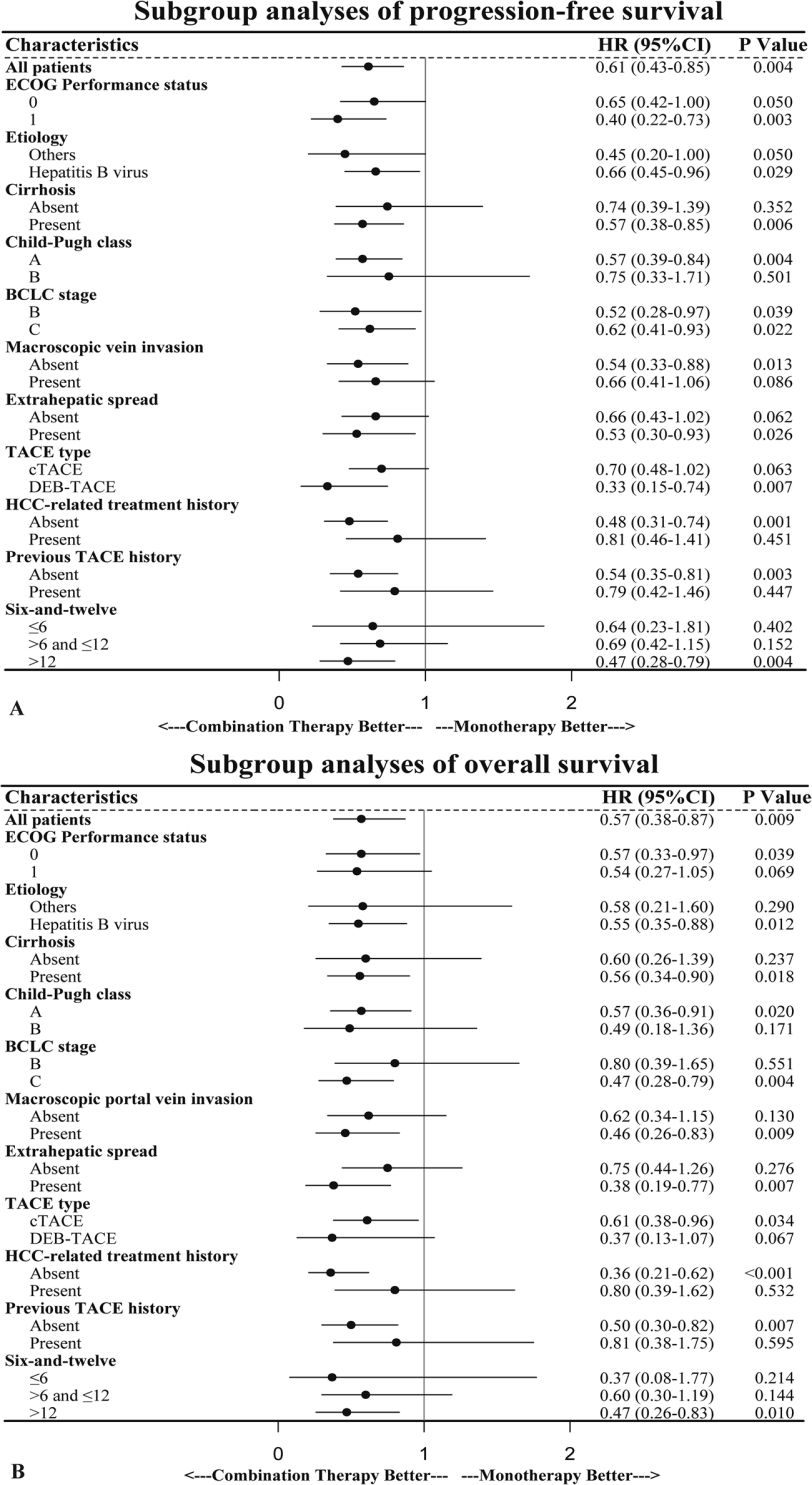
Subgroup analysis of progression-free survival (**A**) and overall survival (**B**) after matching. HR, hazard ratio; CI, confidence interval; ECOG, Eastern Cooperative Oncology Group; BCLC, Barcelona Clinic Liver Cancer; TACE, transarterial chemoembolization; cTACE, conventional transarterial chemoembolization; DEB-TACE, drug-eluting beads transarterial chemoembolization; HCC, hepatocellular carcinoma

**Fig. 4:**
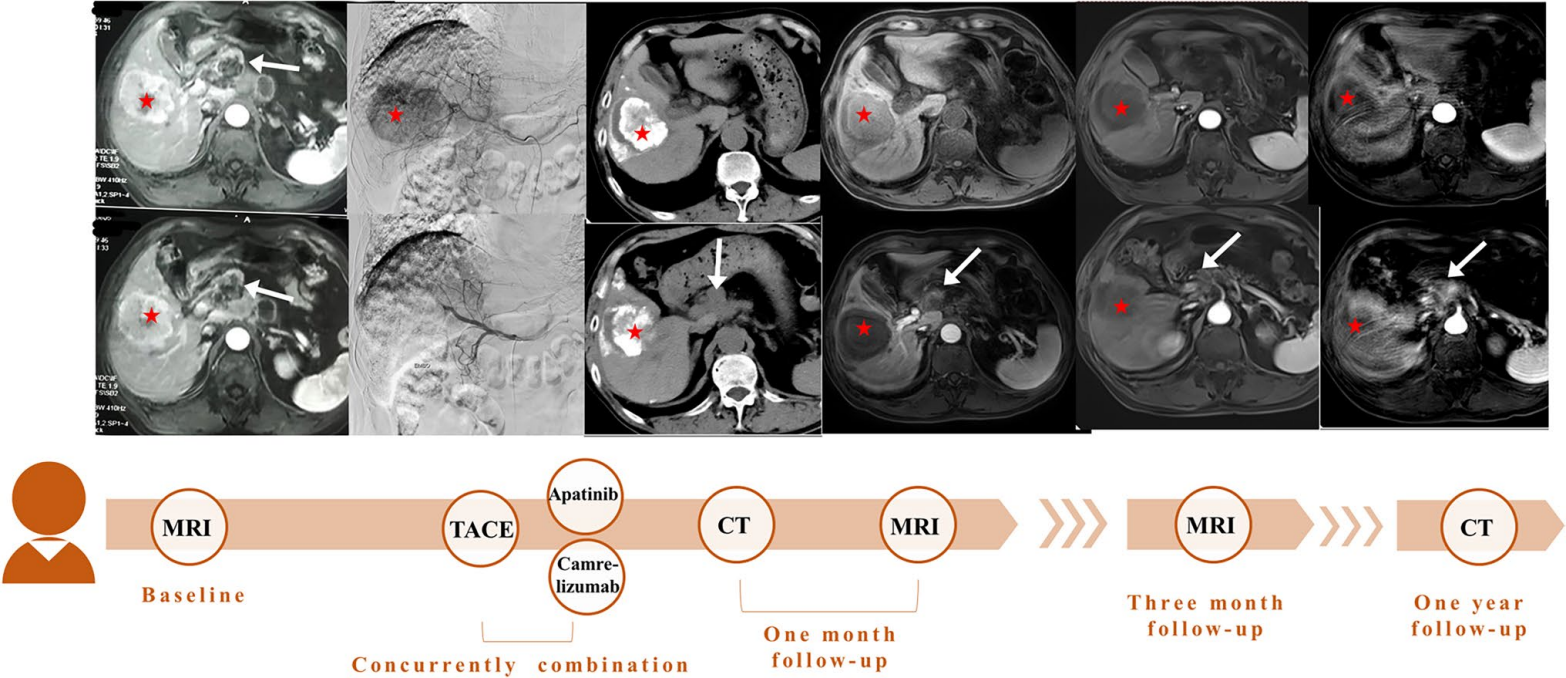
A 72-year-old man had a history of chronic hepatitis B for more than twenty years. The baseline MRI showed that there is a typical HCC lesion (red asterisk) in the right liver lobe with intrahepatic metastasis. The enlargement of the porta hepatic lymph node (white arrow) was considered an extrahepatic spread malignancy. Then, the patient received TACE combined with camrelizumab (200 mg, ivgtt, Q3w) and apatinib (250 mg, po, QD). The first, three-month, 1-year follow-up imaging after combination therapy showed a partial response. MRI, magnetic resonance imaging; CT, computed tomography; HCC, hepatocellular carcinoma; TACE, transarterial chemoembolization

Multivariable analysis, after adjusting potential confounders, showed that combination therapy was significantly associated with longer PFS (adjusted hazard ratio [HR] for progression or death, 0.52; 95% CI, 0.37–0.74; *p* < 0.001) and longer OS (adjusted HR for death, 0.41; 95% CI, 0.26–0.64; *p* < 0.001) than monotherapy in the matched cohorts (Table 2). The multivariable Cox regression analysis in the unmatched cohorts also demonstrated a similar result (Table S1). Subgroup analysis showed a trend that persisted in longer PFS and OS benefits with combination therapy compared to the monotherapy group (Fig. 3 , 4, and Fig. S4). The results of four sensitivity analyses confirmed that combination therapy was associated with significantly better PFS and OS (detailed in supplementary appendix pages 3–4).

**Table 2:** Predictors of progression-free survival and overall survival after matching

	Univariable analysis			Multivariable analysis		
	HR	95% CI	*p* value	HR	95% CI	*p* value
PFS analyses						
ECOG PS (1 *vs.* 0)	1.41	1.01–1.96	0.043	1.35	0.96–1.91	0.085
Etiology (HBV *vs.* others)	1.27	0.87–1.85	0.215			
Cirrhosis (present *vs.* absent)	0.88	0.62–1.25	0.461			
Child-Pugh class (B *vs.* A)	1.91	1.25–2.92	0.003	1.63	1.03–2.59	0.038
BCLC stage (C *vs.* B)	1.53	1.09–2.14	0.013	0.87	0.44–1.70	0.682
Six-to-twelve (> 12 vs. > 6 & ≤ 12 vs. ≤ 6)	1.66	1.30–2.11	< 0.001	1.52	1.17–1.97	0.002
Macroscopic vein invasion (present *vs.* absent)	1.64	1.19–2.25	0.002	1.49	0.86–2.57	0.152
Extrahepatic spread (present *vs.* absent)	1.35	0.98–1.88	0.068	1.21	0.74–2.00	0.451
TACE type (DEB-TACE *vs.* cTACE)	1.02	0.70–1.48	0.934			
HCC-related treatment history (present *vs.* absent)	0.63	0.46–0.88	0.007	0.86	0.59–1.25	0.420
Previous TACE history (present *vs.* absent)	0.78	0.55–1.11	0.169			
Treatment (combination therapy *vs.* monotherapy)	0.61	0.43–0.85	0.004	0.52	0.37–0.74	< 0.001
OS analyses						
ECOG PS (1 *vs.* 0)	1.47	0.98–2.19	0.061	1.28	0.85–1.94	0.236
Etiology (HBV *vs.* others)	1.33	0.82–2.15	0.244			
Cirrhosis (present *vs.* absent)	1.09	0.70–1.70	0.704			
Child-Pugh class (B *vs.* A)	2.10	1.27–3.47	0.004	2.29	1.32–3.98	0.003
BCLC stage (C *vs.* B)	1.50	1.00–2.27	0.052	0.64	0.34–1.22	0.176
Six-to-twelve (> 12 vs. > 6 & ≤ 12 vs. ≤ 6)	1.97	1.45–2.67	< 0.001	1.68	1.20–2.34	0.002
Macroscopic vein invasion (present *vs.* absent)	1.92	1.30–2.84	0.001	2.19	1.20–3.97	0.010
Extrahepatic spread (present *vs.* absent)	1.40	0.94–2.09	0.102			
TACE type (DEB-TACE *vs.* cTACE)	0.87	0.53–1.43	0.573			
HCC-related treatment history (present *vs.* absent)	0.47	0.31–0.71	< 0.001	0.43	0.19–0.95	0.037
Previous TACE history (present *vs.* absent)	0.64	0.41–0.99	0.047	1.44	0.64–3.24	0.378
Treatment (combination therapy *vs.* monotherapy)	0.57	0.38–0.87	0.009	0.41	0.26–0.64	< 0.001

The multivariable analysis includes the variables with *p*-value  ≤ 0.1 from the univariable analysis. *HR*, hazard ratio; *CI*, confidence intervals; *ECOG PS*, Eastern Cooperative Oncology Group performance status; *HBV*, hepatitis B virus; *BCLC*, Barcelona Clinic Liver Cancer; *TACE*, transarterial chemoembolization; *cTACE*, conventional TACE; *DEB-TACE*, drug-eluting beads TACE; *HCC*, hepatocellular carcinoma

### Safety

A total of 117/231 (50.6%) of all patients had a treatment-emergent adverse event from any cause, with higher rates in the combination therapy group (51/84 [60.7%] patients with combination therapy vs. 66/147 [44.9%] patients with TACE monotherapy; Table 3). The most common adverse events in the combination group were aspartate aminotransferase increase (38/84 [45.2%] patients with TACE plus camrelizumab and apatinib vs. 29/147 [19.7%] patients with TACE monotherapy) followed by abdominal pain (34/84 [40.5%] vs. 46/147 [31.3%]), alanine aminotransferase increased (33/84 [39.3%] vs. 25/147 [17.0%]). Grade 3 or 4 AEs were observed in 14 (16.7%) of the 84 patients who received combination therapy, and 12 (8.2%) of 147 patients in the monotherapy group. No grade 5 AEs were observed in whole cohorts. There were 4/84 (4.8%) patients with the discontinuation of camrelizumab and 10/84 (11.9%) patients with the discontinuation of apatinib due to AEs. Dose interruption of camrelizumab occurred in 7/84 (8.3%) patients. Eight (9.5%) of 84 patients suffered from dose reduction or interruption of apatinib. Detailed AE profiles are summarized in the supplementary (Table S2-S3).

**Table 3:** Adverse events from any cause after matching

	Combination group (*n* = 84)	Monotherapy group (*n* = 147)
Patients with an adverse event from any cause	51 (60.7)	66 (44.9)
Grade 1 or 2 event*	37 (44.0)	54 (36.7)
Grade 3 or 4 event*	14 (16.7)	12 (8.2)
Grade 5 event	0	0
Discontinuation of camrelizumab^#^	4 (4.8)	N/A
Discontinuation of apatinib^#^	10 (11.9)	N/A
Dose interruption of camrelizumab	7 (8.3)	N/A
Dose reduction or interruption of apatinib	8 (9.5)	N/A

Data are *n* (%). * Numbers represent the highest grades assigned. ^#^Drug was discontinued due to unacceptable toxic effects. *N/A*, not applicable

## Discussion

This multicenter, retrospective cohort study of patients with predominantly advanced HCC, showed that TACE plus camrelizumab and apatinib is associated with a higher PFS, OS, and ORR than TACE monotherapy. Grade 3 or 4 adverse events occurred in 14/84 (16.7%) patients and 12/147 (8.2%) in the combination and monotherapy groups. These findings were consistently substantiated by the entire cohorts or propensity-score matched cohorts. Multivariable analysis which included potential confounders identified that combination therapy is the independent predictor for longer PFS and OS. This improvement was also consistently observed across all sensitivity and subgroup analyses for survival.

TACE controls tumor lesions by blocking the feeding arteries of the lesions to induce ischemic and hypoxic changes [18, 32, 33]. TACE could enhance PD-1 and PD-L 1 expression in HCC, and induce tumor cell death with the release of tumoral antigens, proinflammatory cytokines, VEGF, and HIF-1α [18–20]. Pinato et al analyzed 119 patients who underwent surgery with or without prior TACE treatment and found that TACE is associated with lower post-treatment intratumoral CD8 + /PD-1 + and T-regs with significant upregulation of pro-inflammatory pathways[19]. Notably, the median interval from the last TACE to surgery was 3.4 months which was consistent with the timeframe criteria defined in this study. These effects could transform an immunosuppressive “cold tumor” into an immunosupportive “hot tumor,” which may enhance the immune response of immune checkpoint inhibitors.

Several previous randomized controlled trials were aimed at investigating potential synergies between TACE and tyrosine kinase inhibitors, such as sorafenib, brivanib, or orantinib with mostly negative results [34]. Although TACTICS was the first positive trial of TACE in combination with sorafenib, there was a time-to-unTACEable progression endpoint based on a novel response assessment criterion that needs to be tested and validated in a further large-scale randomized controlled trial [7, 18]. To date, the clinical evidence fails to support the use of TACE in combination with tyrosine kinase inhibitors not only for intermediate HCC but also for advanced HCC [35]. Recently, the combination of atezolizumab (anti-PD-L1 antibody) and bevacizumab (anti-angiogenesis agent), the IMbrave150 study, and the combination of sintilimab (anti-PD-1 antibody) and a bevacizumab biosimilar, the ORIENT-32 trial, have successfully outperformed sorafenib as the first-line therapy in advanced HCC [9, 10]. The favorable advances pose this combination of immune checkpoint inhibitors with molecularly targeted therapies as the recommended systemic therapy protocol and lead to treatment paradigm changes [36]. Camrelizumab plus apatinib showed promising antitumor activity in advanced HCC from the results of several prospective trials. Currently, the combination of camrelizumab and apatinib has been available and is included in the basic medical insurance system of China.

Our study found that TACE combined with camrelizumab (anti-PD-1 antibody) plus apatinib (tyrosine kinase inhibitors) was associated with better efficacy. The median PFS was 13.5 months (95%CI, 9.0–18.0) with an ORR of 59.5% (50/84) after matching. In the phase II RESCUE trial, the median PFS was 5.7 months (95% CI, 5.4–7.4) with an ORR of 45.7% (95%CI, 33.7–58.1) in the first-line setting, and 5.5 months (95% CI, 3.7–5.6) with 25.0% (95% CI, 17.5–33.7) in the second-line setting [15]. The recently reported phase III trial demonstrated that camrelizumab plus apatinib has a median PFS of 5.6 months (95% CI 5.5–6.3) with an ORR of 25.4% (20.3–31.0), which was consistent with phase II RESCUE trial [15, 16]. When compared with IMbrave 150 and ORIENT-32 trials (PFS, 6.8 and 4.6 months; ORR, 35.4% and 24.3% according to mRECIST), the longer PFS and higher ORR were achieved in the combination group [9, 10, 37]. The median OS of patients treated with TACE plus camrelizumab and apatinib was 24.1 months (95%CI, 20.0–NR), which was higher than the updated IMbrave 150 trial data (19.2 months [95% CI,17.0–23.7]), and the phase III trial of camrelizumab plus apatinib (22.1 months [95% CI 19.1–27.2]) [16, 37]. In terms of safety, patients with an adverse event from any cause were 51/84 (60.7%), and grade 3 or 4 adverse events were 14/84 (16.7%) in the combination group. Reactive cutaneous capillary endothelial proliferation occurred in 9 (10.7%) of 84 patients. Approximately 4.8% of patients discontinued camrelizumab and 11.9% of patients discontinued apatinib. In the RESCUE trial, grade  ≥ 3 treatment-related AEs were reported in 147 (77.4%) patients, with two treatment-related deaths occurring [15]. The most common AEs were hypertension (34.2%), and reactive cutaneous capillary endothelial proliferation occurred in 56 (29.5%) patients. In the phase III trial of camrelizumab plus apatinib, grade  ≥ 3 treatment-related AEs occurred in 80.9% versus. 52.4% with sorafenib. Treatment-related AEs led to discontinuation of any treatment is 24.3% (of both agents 3.7%) with camrelizumab plus apatinib.

Based on the above data, TACE combined with systemic therapy seem to provide better results than systemic therapy alone. There were several possible causes for these survival benefits, besides the potential synergistic enhancement from TACE treatment. TACE has the advantage of rapid response, while the advantages of systemic therapy are in the extension and durability of the antitumor response [18, 38, 39]. The predominant etiology of HCC was the hepatitis B virus in this study, which could be more likely to benefit from immunotherapy [40]. Also, the patients included in this study have an earlier tumor stage and better performance status compared to clinical trials. However, these survival benefits need to be validated in the prospective clinical trials. Further investigations of synergistic enhancement effects for TACE in HCC patients treated with systemic therapy are warranted.

There are some limitations in this study. First, this is a retrospective study with a small sample size. However, the PSM method and several sensitivity analyses were used to reduce potential selection bias as much as possible. Recall bias is a potential limitation in all patient–control studies and it possibly affected adverse effects assessment. Third, this study included predominant HBV-related HCC patients in China. It remains to be elucidated whether the efficacy of combination therapy could be widely applied in patients with other etiologies. The fourth limitation is the lack of sample size calculation before starting the study. Nevertheless, the result of the power calculation shows that the study power is over 90%, which is the probability of rejecting a false null hypothesis. Another limitation is the heterogeneity in TACE procedure, not only between real-world clinical practice and clinical trials but also in different regions (such as European) with different practices [41]. The standardization of TACE procedures across participating centers could to some extent minimize this heterogeneity [26, 27]. Additionally, the ORR of TACE monotherapy was 37.4% before matching and 39.2% after matching, which was relatively low than the expected outcomes reported in previous trials, ranging from 45 to 54% [42]. This discrepancy may be attributed to more patients included in this study with severe performance status and liver function, advanced disease stage, and higher tumor burden.

In conclusion, compared to TACE monotherapy, TACE plus camrelizumab and apatinib provides OS, PFS, and ORR benefits for intermediate and advanced HCC patients, with a higher incidence of adverse events. The efficacy of this combination therapy still needs to be further validated in prospective, randomized trials.

### Supplementary Information

Below is the link to the electronic supplementary material.

Supplementary file1 (PDF 499 KB)

## Abbreviations

CI: Confidence interval
HCC: Hepatocellular carcinoma
HR: Hazard ratio
mRECIST: Modified Response Evaluation Criteria in Solid Tumors
ORR: Objective response rate
OS: Overall survival
PD-(L)1: Programmed death-(ligand) 1
PFS: Progression-free survival
TACE: Transarterial chemoembolization

## Declarations

### Guarantor

The scientific guarantor of this publication is Gao-Jun Teng.

### Statistics and biometry

No complex statistical methods were necessary for this paper.

### Methodology

• retrospective

• observational

• multicenter study
