# Supplementary material for: Real-world efficacy and safety of TACE plus camrelizumab and apatinib in patients with HCC (CHANCE2211): a propensity score matching study
Source: Eur Radiol. 2023 Jun 27;33(12):8669–81. doi: 10.1007/s00330-023-09754-2 (PMC10667391; doi:10.1007/s00330-023-09754-2)
Supplement: Supplementary file 1 — Supplementary file1 (PDF 499 KB) [file 330_2023_9754_MOESM1_ESM.pdf]

ELECTRONIC SUPPLEMENTARY MATERIAL

Real-World Efficacy and Safety of TACE plus Camrelizumab and Apatinib in Patients with HCC (CHANCE2211): A Propensity Score Matching Study

This appendix has been provided by the authors to give readers additional information about their work.

Contents of Supplementary Appendix

Contents	Page
Detailed Transarterial Chemoembolization Procedure Protocol	2
Sensitivity analyses	3-4
Power calculation	4
Table S1. Predictors of progression-free survival and overall survival before matching.	5-6
Table S2: Treatment-related adverse events after matching	7
Table S3. Adverse events in two cohorts after matching	8
Figure S1. Absolute standardized mean difference of propensity score matching	9
Figure S2. Kaplan–Meier analysis of progression-free survival before matching	10
Figure S3. Kaplan–Meier analysis of overall survival before matching	11
Figure S4. Subgroup analysis of progression-free survival and overall survival before matching	12
Reference	13

Methods

Detailed Transarterial Chemoembolization Procedure Protocol

Patients included in the study received conventional TACE (cTACE) or drug-eluting beads TACE (DEB-TACE). All the TACE procedures were applied according to standardization protocols in the all-participated hospital[1-3]. Adequate visualization of all tumor-feeding feeding arteries should be obtained during the procedure, including vessels' origin, variant anatomy, and ectopic or collateral blood supply. Feeding arteries of the tumors were as selective as possible in order to obtain better treatment efficacy and to reduce treatment-related complications both for cTACE and DEB-TACE. The endpoint of TACE is defined as a "tree in winter" appearance in case of non-selective TACE.

For cTACE, an emulsion of mixtures of lipiodol (2-20 ml) and chemotherapeutic drugs was injected to the feeding arteries of the tumors. Doxorubicin is the most common single chemotherapeutic drug. The dosage of chemotherapeutic drug used could be body surface area-based, liver function-based, weight-based, or even empiric. Chemotherapeutic drugs including doxorubicin (10-100 mg), epirubicin (5-120mg), oxaliplatin (100-200 mg), cisplatin (10-100 mg) and other drugs were selected according to clinical practice of the participating centers. The ethiodized oil and chemotherapeutic drugs should be mixed into an emulsion and configured as a "water-in-oil" emulsifier to improve its stability. The volume ratio of ethiodized oil to drug aqueous solution is usually 2:1. The volume of ethiodized oil injected is generally determined by the size and vascularity of the tumor, with common usage of 5–15 mL. Finally, particulate embolic agents (e.g., standardized gelatin sponge particles, microspheres, polyvinyl alcohol particles) should be used following embolization with ethiodized oil chemoembolic emulsion to achieve a satisfied embolization endpoint.

For DEB-TACE, a dose of 2-4 ml DC beads (Biocompatibles, Farnham, United Kingdom) or Callispheres beads (Jiangsu Hengrui Medicine Co., Ltd., Jiangsu, China) or HepaSpheres beads (Biosphere Medical, Inc., South Jordan, UT) with a diameter of 100-300 or 300-500 μm loaded with epirubicin (with a maximum dose of 100 mg) were introduced. Additional embolization was applied if satisfied embolization endpoint was not achieved.

"On demand" TACE procedures were repeated based on the demonstration of viable tumors or intrahepatic recurrences by contrast-enhanced computed tomography (CT) or magnetic resonance imaging (MR). All TACE procedures were performed by physicians with at least 10 years of experience on interventional radiology from participating centers. When residual viable tumors were confirmed or new lesions developed in patients with adequate liver function, repeated TACE was performed.

TACE was discontinued if one of the following conditions occurred: 1) deterioration of liver function to Child-Pugh C (uncontrollable ascites, severe jaundice, overt hepatic encephalopathy, or hepatorenal syndrome); 2) Eastern Cooperative Oncology Group (ECOG >2); 3) continued progression of target lesions after 3 TACE sessions according to clinical practice of the participating centers.

Sensitivity analyses

We conducted four sensitivity analyses to assess the robustness of the propensity score matching (PSM) analyses. First, we used one to two optimal matching method with the same variables as described in manuscript. Propensity scores were calculated using a logistic regression model by the following variables: sex, age, ECOG performance status, hepatitis B virus, cirrhosis, Child-Pugh grade, six-and-twelve criteria, BCLC stage, macroscopic vein invasion, extrahepatic spread, and HCC-related treatment history. After matching, 107 and 214 patients remained in each group. The median PFS was 11.6 months (95% CI, 9.7 to 15.5) in the combination group, which was significantly longer than that in the monotherapy group (7.7 months [95% CI, 6.6 to 9.3]; $p < 0.001$). The median OS was 24.1 months (95% CI, 19.5 to NR) with ORR 55.1% in the combination group, which was significantly longer than that in the monotherapy group (15.7 months [95% CI, 14.1 to 18.7]; $p = 0.002$; ORR, 32.7%, $p < 0.001$). After adjusted the covariates, multivariable Cox regression analysis showed that combination therapy (for PFS, hazard ratio [HR] 0.59; 95% CI 0.44 to 0.80; $p = 0.001$; for OS, HR 0.51; 95% CI 0.35 to 0.74; $p < 0.001$) were the independent prognostic indicators in all patients weighting analysis cohort.

Second, PSM analysis was performed and 1:2 nearest-neighbor matching without replacement using a caliper width of 0.05 was set. The up-to-seven criteria (≤7 vs. >7) were included as covariates and replaced the six-and-twelve criteria. Other covariates were included, as follows: sex, age, ECOG performance status, HBV infection (absent vs. present), cirrhosis (absent vs. present), Child-Pugh grade (A vs. B), BCLC stage (B vs. C), extrahepatic spread (absent vs. present), macroscopic vein invasion (absent vs. present), and HCC-related treatment history (absent vs. present). After matching, 85 and 154 patients remained in combination group and monotherapy group, respectively. The median PFS was 12 months (95% CI, 9.1 to 16.6) in the combination group, which was significantly longer than that in the monotherapy group (9 months [95% CI, 6.9 to 11.2]; $p = 0.02$). The median OS was 23.9 months (95% CI, 19.5 to NR) with ORR 56.5% in the combination group, which was significantly longer than that in the monotherapy group (16.1 months [95% CI, 14.1 to 24.9]; $p = 0.04$; ORR, 31.8%, $p < 0.001$). After adjusting the covariates, multivariable Cox regression analysis showed that combination therapy (for PFS, adjusted HR 0.60; 95% CI 0.42 to 0.85; $p = 0.004$; for OS, adjusted HR 0.53; 95% CI 0.35 to 0.82; $p = 0.004$) were the independent prognostic indicators.

Then, we performed PSM with several key clinical factors (ECOG performance status, hepatitis B virus, Child-Pugh grade, and BCLC stage), which indicated patients' general well-being, etiology, liver function, and tumor stage. On the other hand, these excluded factors (sex, age, cirrhosis, macroscopic vein invasion, extrahepatic spread, and HCC-related treatment history) were either balanced before matching, or not definitive prognostic for PFS/OS in the previous study, or associated with the above four factors. The 1:2 nearest-neighbor method with caliper widths of 0.05 was used.

After matching, 107 and 205 patients remained in each group. There were small differences remained for other characteristics not included in the PSM. The median PFS was 11.6 months (95% CI, 9.7 to 15.5) in the combination group, which was significantly longer than that in the monotherapy group (8.9 months [95% CI, 7.1 to 10.3]; $p = 0.003$). The median OS was 24.1 months (95% CI, 19.5 to NR) with ORR 55.1% in the combination group, which was significantly longer than that in the monotherapy group (16.6 months [95% CI, 13.6 to 22.7]; $p = 0.01$; ORR, 35.6%, $p = 0.001$). After adjusted the covariates, multivariable Cox regression analysis showed that combination therapy (for PFS, HR 0.50; 95% CI 0.36 to 0.70; $p < 0.001$; for OS, HR 0.52; 95% CI 0.35 to 0.77; $p = 0.001$) were the independent prognostic indicators in all patients weighting analysis cohort.

For the inverse probability of treatment weighting analysis, we calculated the probability of receiving the combination therapy (propensity score) for each patient using a logistic regression model. The model included the following variables: sex, age, ECOG performance status, hepatitis B virus, cirrhosis, Child-Pugh grade, up-to-seven criteria, BCLC stage, macroscopic vein invasion, extrahepatic spread, and HCC-related treatment history. We calculated individual weights using the propensity score as follows: 1/propensity score for patients receiving the combination therapy, and 1/(1–propensity score) for monotherapy. The median PFS was 13.5 months (95% CI, 9.7 to 18.0) in the combination group, which was significantly longer than that in the monotherapy group (8.8 months [95% CI, 7.7 to 9.8]; $p = 0.005$). The median OS was 26.1 months (95% CI, 20.0 to NR) in the combination group, which was significantly longer than that in the monotherapy group (16.9 months [95% CI, 15.5 to 19.1]; $p = 0.003$). After adjusted the covariates, multivariable Cox regression analysis showed that combination therapy (for PFS, hazard ratio [HR] 0.51; 95% CI 0.36 to 0.72; $p < 0.001$; for OS, HR 0.44; 95% CI 0.30 to 0.67; $p < 0.001$) were the independent prognostic indicators in all patients weighting analysis cohort.

Power calculation

For power calculation, the sample size for two groups were set according to finally cases after PSM (N1=147, N2=84), and median OS for TACE monotherapy was set as 15.7 months in this study (Figure 2). The hazard ratio for combination therapy was set as 0.41 (Table 2). According to study design, the total time was set as 52 months, with follow-up time of 12 months. Finally, A two-sided log rank test with an overall sample size of 231 subjects (147 in the control group and 84 in the treatment group) achieves 99.8% power to detect a hazard ratio of 0.41 at a 0.050 significance level. The result show that the present study power over 90%, which is the probability of rejecting a false null hypothesis. Above power calculation were performed using PASS (version 15.0.5).

Table S1. Predictors of progression-free survival and overall survival before matching

	Univariable analysis			Multivariable analysis		
	HR	95% CI	*p* value	HR	95% CI	*p* value
**PFS analyses**						
**ECOG PS** (1 *vs.* 0)	1.24	0.99-1.56	0.064	1.20	0.95-1.53	0.133
**Etiology** (HBV *vs.* others)	1.14	0.90-1.43	0.279			
**Cirrhosis** (present *vs.* absent)	0.99	0.81-1.22	0.930			
**Child-Pugh class** (B *vs.* A)	1.30	0.98-1.72	0.065	1.08	0.81-1.45	0.585
**BCLC stage** (C *vs.* B)	1.68	1.37-2.05	<0.001	1.29	0.82-2.01	0.269
**Six-to-twelve** (≤6 vs. <6&≤12 vs. >12)	1.53	1.31-1.78	<0.001	1.44	1.23-1.69	<0.001
**Macroscopic vein invasion** (present *vs.* absent)	1.62	1.32-1.98	<0.001	1.19	0.81-1.75	0.367
**Extrahepatic spread** (present *vs.* absent)	1.48	1.18-1.84	0.001	1.36	0.98-1.88	0.063
**TACE type** (DEB-TACE *vs.* cTACE)	0.99	0.78-1.25	0.923			
**HCC-related treatment history** (present *vs.* absent)	0.71	0.56-0.90	0.005	0.86	0.56-1.31	0.484
**Previous TACE history** (present *vs.* absent)	0.78	0.59-1.02	0.066	1.09	0.68-1.74	0.725
**Treatment** (combination therapy *vs.* monotherapy)	0.68	0.52-0.89	0.005	0.55	0.41-0.73	<0.001
**OS analyses**						
**ECOG PS** (1 *vs.* 0)	1.19	0.90-1.57	0.216			
**Etiology** (HBV *vs.* others)	1.06	0.80-1.40	0.701			
**Cirrhosis** (present *vs.* absent)	0.99	0.77-1.28	0.963			
**Child-Pugh class** (B *vs.* A)	1.76	1.28-2.42	0.001	1.50	1.08-2.08	0.016
**BCLC stage** (C *vs.* B)	1.67	1.30-2.15	<0.001	0.80	0.46-1.38	0.418
**Six-to-twelve** (≤6 vs. <6&≤12 vs. >12)	1.74	1.44-2.10	<0.001	1.57	1.29-1.90	<0.001
**Macroscopic vein invasion** (present *vs.* absent)	1.87	1.46-2.40	<0.001	1.99	1.24-3.20	0.004
**Extrahepatic spread** (present *vs.* absent)	1.45	1.11-1.91	0.007	1.76	1.20-2.59	0.004
**TACE type** (DEB-TACE *vs.* cTACE)	0.94	0.69-1.27	0.667			
**HCC-related treatment history** (present *vs.* absent)	0.54	0.40-0.73	<0.001	0.62	0.35-1.11	0.110
**Previous TACE history** (present *vs.* absent)	0.65	0.46-0.91	0.013	1.26	0.67-2.38	0.475
**Treatment** (combination therapy *vs.* monotherapy)	0.61	0.44-0.85	0.003	0.49	0.34-0.70	<0.001

The multivariable analysis includes the variables with *p*-value≤0.1 from the univariable analysis. HR, hazard ratio; CI, confidence intervals; ECOG PS, Eastern

Cooperative Oncology Group performance status; HBV, hepatitis B virus; BCLC, Barcelona Clinic Liver Cancer; TACE, transarterial chemoembolization; cTACE, conventional TACE; DEB-TACE, drug-eluting beads TACE; HCC, hepatocellular carcinoma.

Table S2: Treatment-related adverse events after matching

Variable	Combination group (n = 84)	Monotherapy group (n = 147)
TACE-related event	35 (41.7)	66 (44.9)
Grade 1 or 2 event*	30 (35.7)	54 (36.7)
Grade 3 or 4 event*	5 (6.0)	12 (8.2)
Grade 5 event*	0	0
Camrelizumab-related event	25 (29.8)	N/A
Grade 1 or 2 event*	22 (26.2)	N/A
Grade 3 or 4 event*	3 (3.6)	N/A
Grade 5 event*	0	N/A
Apatinib-related event	27 (32.1)	N/A
Grade 1 or 2 event*	19 (22.6)	N/A
Grade 3 or 4 event*	8 (9.5)	N/A
Grade 5 event*	0	N/A

Data are n (%). * Numbers represent the highest grades assigned. N/A, not applicable.

Table S3. Adverse events in two cohorts after matching

	All grades	Grades 1 or 2	Grade 3 or 4	Grade 5
**Combination Group**				
Increased AST	38 (45.2)	36 (42.9)	2 (2.4)	0
Abdominal pain	34 (40.5)	32 (38.1)	2 (2.4)	0
Increased ALT	33 (39.3)	30 (35.7)	3 (3.6)	0
Pyrexia	23 (27.4)	22 (26.2)	1 (1.2)	0
Elevated bilirubin	16 (19.0)	16 (19.0)	0	0
Hypertension	16 (19.0)	15 (17.9)	1 (1.2)	0
Fatigue	12 (14.3)	9 (10.7)	3 (3.6)	0
Hand-foot skin reaction	11 (13.1)	7 (8.3)	4 (4.8)	0
RCCEP	9 (10.7)	8 (9.5)	1 (1.2)	0
Nausea	7 (8.3)	7 (8.3)	0	0
Vomiting	7 (8.3)	7 (8.3)	0	0
Diarrhea	7 (8.3)	5 (6.0)	2 (2.4)	0
Hypothyroidism	6 (7.1)	6 (7.1)	0	0
Proteinuria	5 (6.0)	3 (3.6)	2 (2.4)	0
Rash	5 (6.0)	5 (6.0)	0	0
Pruritus	2 (2.4)	2 (2.4)	0	0
Hepatitis	2 (2.4)	2 (2.4)	0	0
Thrombocytopenia	1 (1.2)	0	1 (1.2)	0
Hyperthyroidism	1 (1.2)	1 (1.2)	0	0
Headache	1 (1.2)	1 (1.2)	0	0
Laryngeal edema	1 (1.2)	1 (1.2)	0	0
**Monotherapy Group**				
Abdominal pain	46 (31.3)	44 (29.9)	2 (1.4)	0
Increased AST	29 (19.7)	25 (17.0)	4 (2.7)	0
Increased ALT	25 (17.0)	20 (13.6)	5 (3.4)	0
Nausea	24 (16.3)	23 (15.6)	1 (0.7)	0
Vomiting	20 (13.6)	19 (12.9)	1 (0.7)	0
Pyrexia	15 (10.2)	14 (9.5)	1 (0.7)	0
Fatigue	11 (7.5)	10 (6.8)	1 (0.7)	0
Elevated bilirubin	10 (6.8)	9 (6.1)	1 (0.7)	0
Anorexia	7 (4.8)	7 (4.8)	0	0

Data are n (%). The numbers of patients in the two groups after matching are 84 (combination group) and 147 (monotherapy group). AST, aspartate aminotransferase; ALT, alanine aminotransferase; RCCEP, reactive cutaneous capillary endothelial proliferation.

Figure S1. Absolute standardized mean difference of propensity score matching

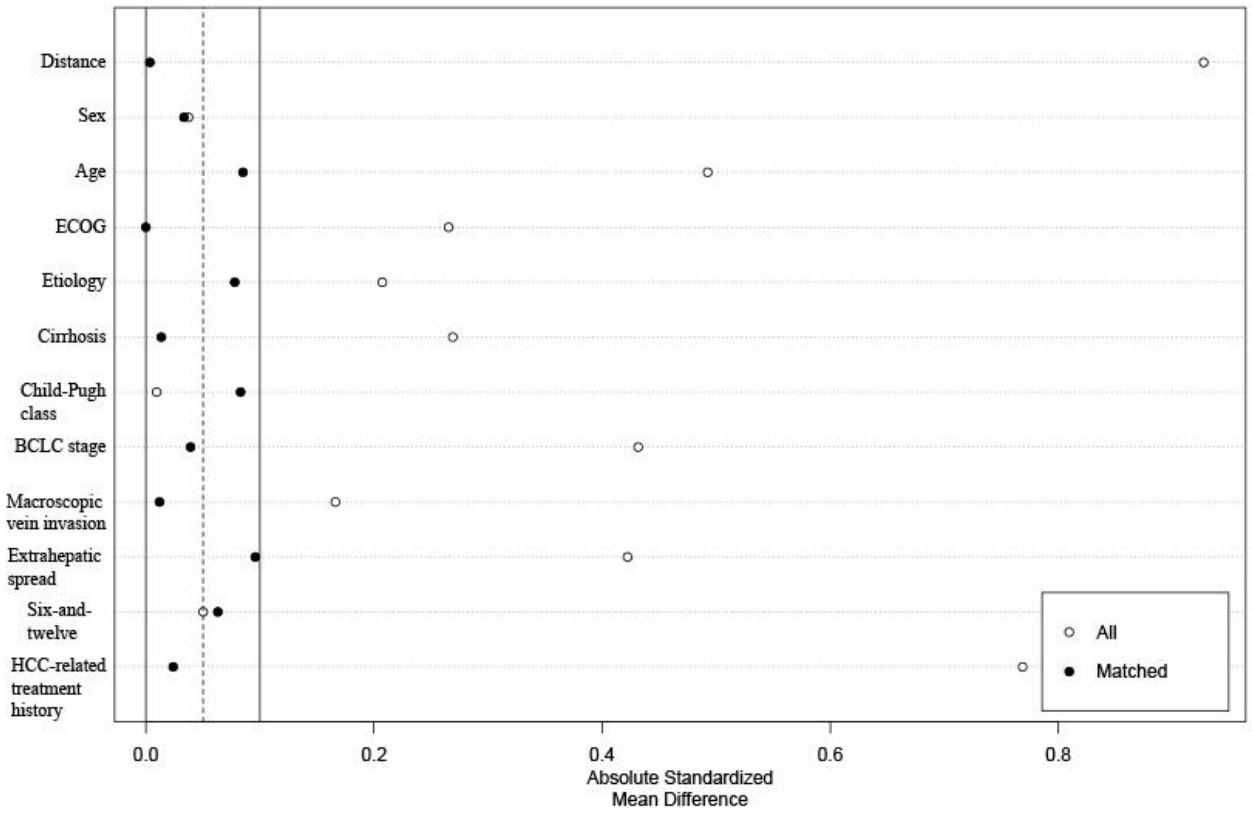

Figure S2. Kaplan–Meier analysis of progression-free survival before matching

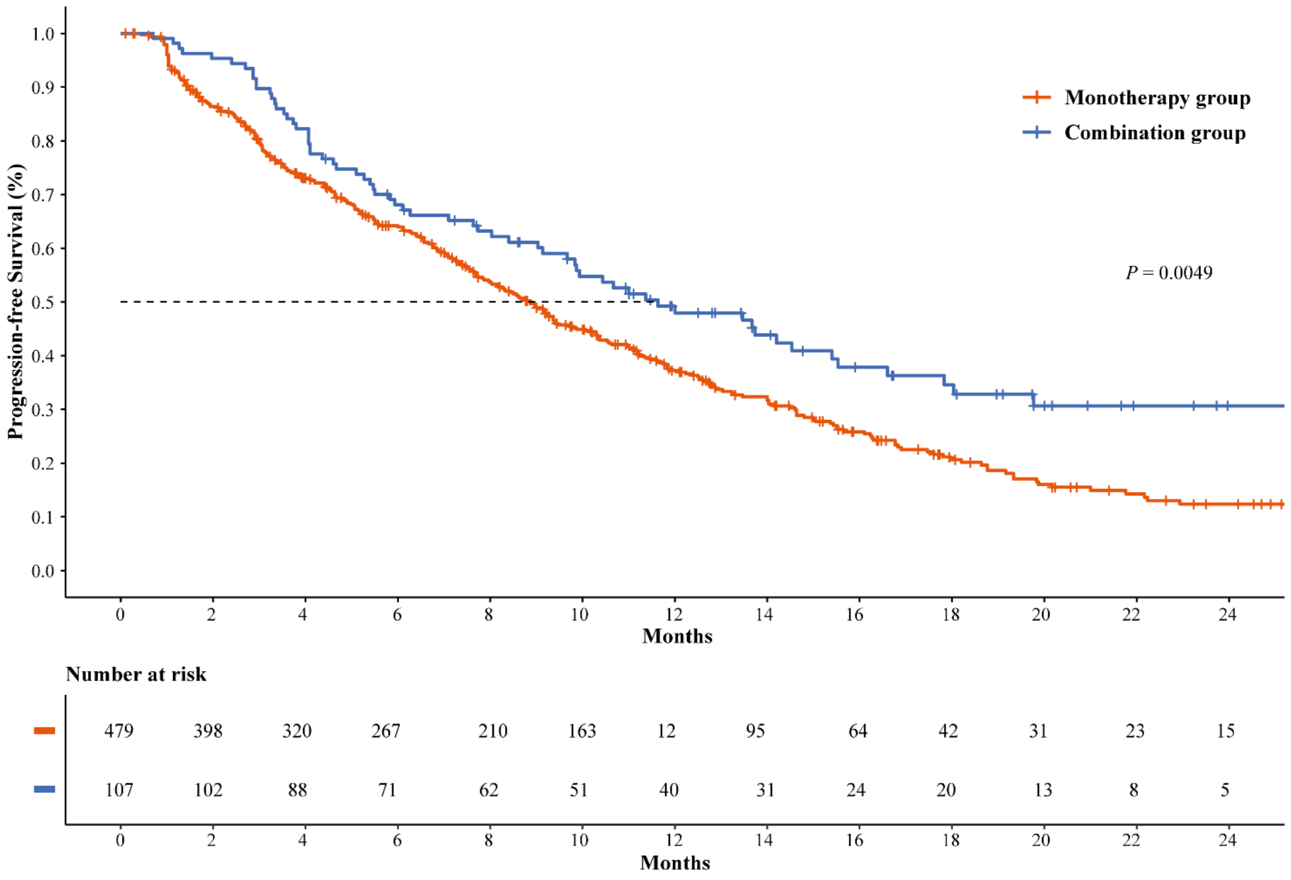

Figure S3. Kaplan–Meier analysis of overall survival before matching

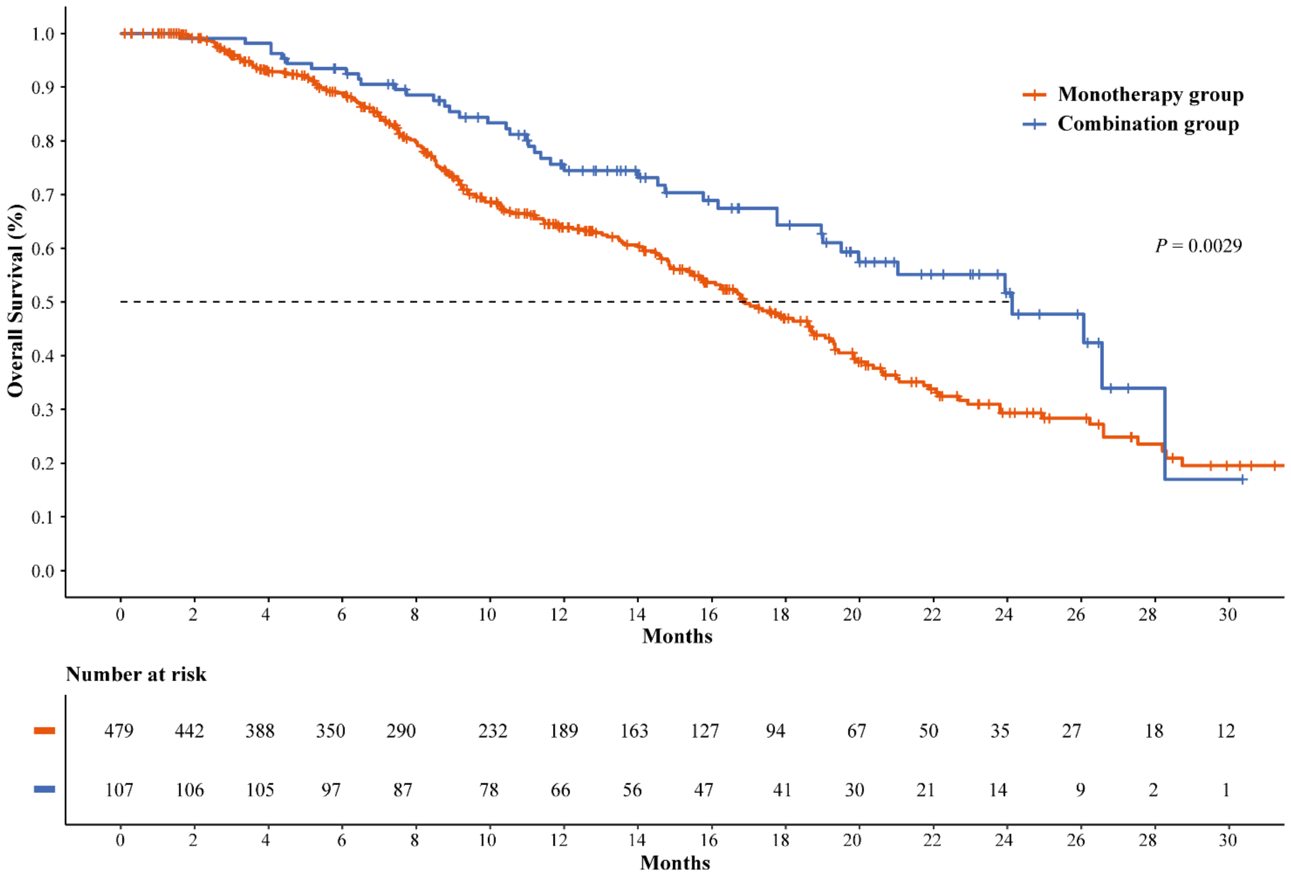

Figure S4. Subgroup analysis of progression-free survival and overall survival before matching

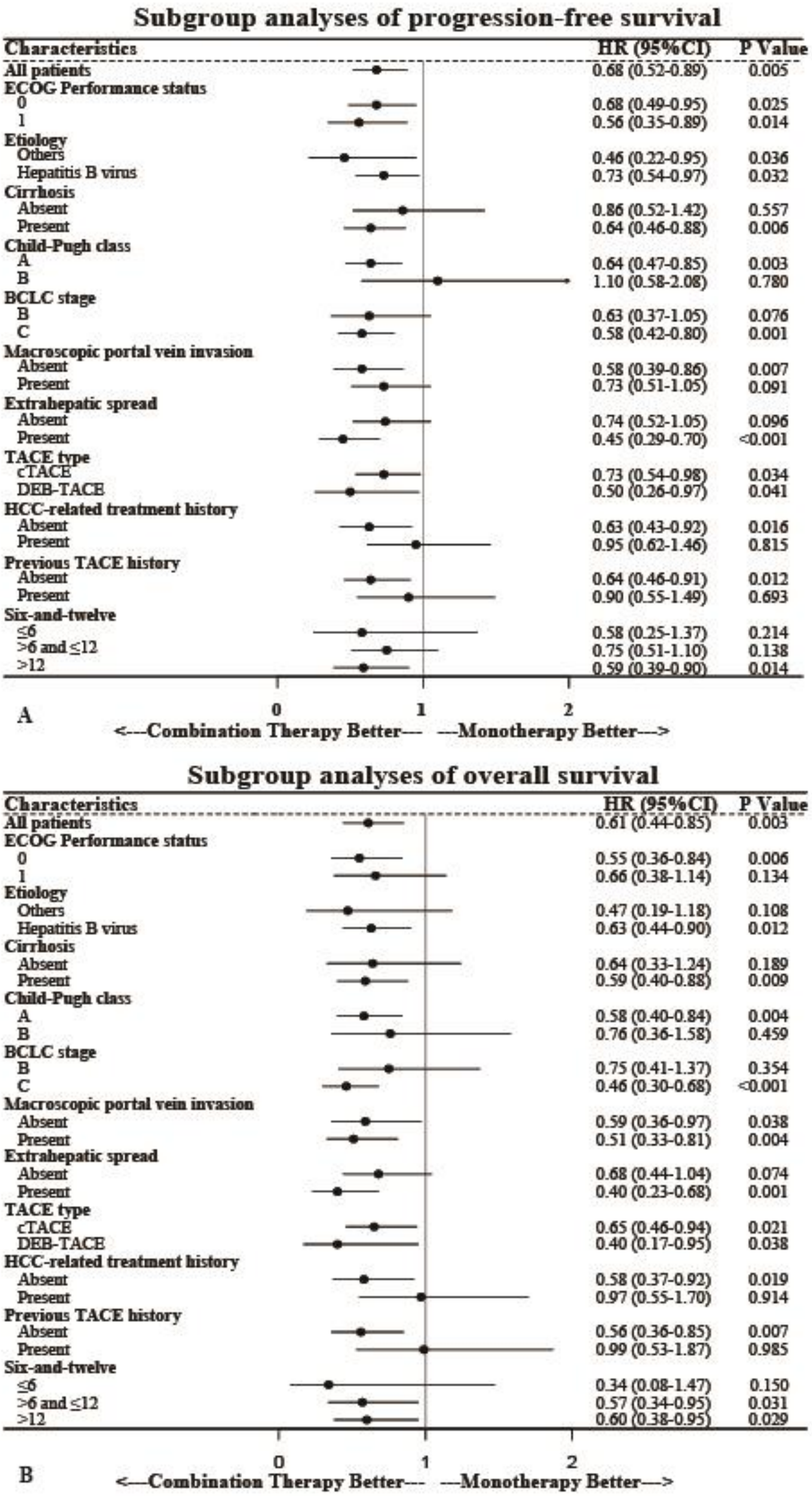

References

1. Galle PR, Forner A, Llovet JM, et al. EASL Clinical Practice Guidelines: Management of hepatocellular carcinoma. *J Hepatol* 2018;69(1):182-236. doi: 10.1016/j.jhep.2018.03.019 [published Online First: 20180405]
2. Marrero JA, Kulik LM, Sirlin CB, et al. Diagnosis, Staging, and Management of Hepatocellular Carcinoma: 2018 Practice Guidance by the American Association for the Study of Liver Diseases. *Hepatology* 2018;68(2):723-50. doi: 10.1002/hep.29913 [published Online First: 2018/04/07]
3. Clinical Guidelines Committee of Chinese Interventionalists C. [Chinese clinical practice guidelines for transarterial chemoembolization of hepatocellular carcinoma]. *Zhonghua Nei Ke Za Zhi* 2021;60(7):599-614. doi: 10.3760/cma.j.cn112137-20210425-00991
